# A versatile strategy for convenient circular bivalent functional nucleic acids construction

**DOI:** 10.1093/nsr/nwac107

**Published:** 2022-06-07

**Authors:** Xiao-Jing Zhang, Zhuo Zhao, Xia Wang, Min-Hui Su, Lili Ai, Yingying Li, Quan Yuan, Xue-Qiang Wang, Weihong Tan

**Affiliations:** Molecular Science and Biomedicine Laboratory (MBL), State Key Laboratory of Chemo/Biosensing and Chemometrics, College of Chemistry and Chemical Engineering, College of Biology, Aptamer Engineering Center of Hunan Province, Hunan University, Changsha 410082, China; Molecular Science and Biomedicine Laboratory (MBL), State Key Laboratory of Chemo/Biosensing and Chemometrics, College of Chemistry and Chemical Engineering, College of Biology, Aptamer Engineering Center of Hunan Province, Hunan University, Changsha 410082, China; Molecular Science and Biomedicine Laboratory (MBL), State Key Laboratory of Chemo/Biosensing and Chemometrics, College of Chemistry and Chemical Engineering, College of Biology, Aptamer Engineering Center of Hunan Province, Hunan University, Changsha 410082, China; Molecular Science and Biomedicine Laboratory (MBL), State Key Laboratory of Chemo/Biosensing and Chemometrics, College of Chemistry and Chemical Engineering, College of Biology, Aptamer Engineering Center of Hunan Province, Hunan University, Changsha 410082, China; Molecular Science and Biomedicine Laboratory (MBL), State Key Laboratory of Chemo/Biosensing and Chemometrics, College of Chemistry and Chemical Engineering, College of Biology, Aptamer Engineering Center of Hunan Province, Hunan University, Changsha 410082, China; Molecular Science and Biomedicine Laboratory (MBL), State Key Laboratory of Chemo/Biosensing and Chemometrics, College of Chemistry and Chemical Engineering, College of Biology, Aptamer Engineering Center of Hunan Province, Hunan University, Changsha 410082, China; Molecular Science and Biomedicine Laboratory (MBL), State Key Laboratory of Chemo/Biosensing and Chemometrics, College of Chemistry and Chemical Engineering, College of Biology, Aptamer Engineering Center of Hunan Province, Hunan University, Changsha 410082, China; Molecular Science and Biomedicine Laboratory (MBL), State Key Laboratory of Chemo/Biosensing and Chemometrics, College of Chemistry and Chemical Engineering, College of Biology, Aptamer Engineering Center of Hunan Province, Hunan University, Changsha 410082, China; Molecular Science and Biomedicine Laboratory (MBL), State Key Laboratory of Chemo/Biosensing and Chemometrics, College of Chemistry and Chemical Engineering, College of Biology, Aptamer Engineering Center of Hunan Province, Hunan University, Changsha 410082, China; Institute of Molecular Medicine (IMM), Renji Hospital, Shanghai Jiao Tong University School of Medicine, and College of Chemistry and Chemical Engineering, Shanghai Jiao Tong University, Shanghai 200240, China; The Cancer Hospital of the University of Chinese Academy of Sciences (Zhejiang Cancer Hospital), Hangzhou Institute of Medicine (HIM), Chinese Academy of Sciences, Hangzhou 310022, China

**Keywords:** circular bivalent aptamers, click chemistry, chemical ligation

## Abstract

Functional nucleic acids (FNAs), such as aptamers, nucleic acid enzymes and riboswitches play essential roles in various fields of life sciences. Tailoring of ingenious chemical moieties toward FNAs can enhance their biomedical properties and/or confer them with exogenic biological functions that, in turn, can considerably expand their biomedical applications, or even improve their clinical translations. Herein, we report the first example of a general chemical tailoring strategy that enables the divergent ligation of DNA sequences. By applying this technology, different types of aptamers and single-stranded nucleic acids of various lengths could be efficiently tailored to deliver the designed circular bivalent aptamers (CBApts) and cyclized DNA sequences with high yields. It is worth noting that CBApts exhibited significantly enhanced nuclease resistance, as well as considerably improved binding, targeting and tumor tissue enrichment abilities, which may pave the way for different investigations for biomedical purposes.

## Teaser

A versatile and efficient nucleic acid ligation strategy was developed to construct different types of circular bivalent aptamers and cyclized DNA sequences that possess significantly enhanced biomedical properties.

## INTRODUCTION

Since the early 1980s when some natural RNA molecules were revealed to function as enzymes, other functional nucleic acids (FNAs) including deoxyribose (DNAzyme) [1,2], aptamer [3,4] and riboswitch [5,6] were subsequently discovered and used in numerous biomedical investigations. In the past few decades, massive advances in FNA techniques have immensely improved our understanding of the dynamic interaction among metabolites, nucleic acids, peptides, proteins and other compounds inside or outside the cells in both normal and diseased states, thus allowing the development of innovative therapeutic agents.

Aptamers, known as chemical antibodies, are among the most attractive FNAs that can specifically recognize various targets and are widely applied in various fields such as bioanalysis, diagnosis and therapeutics [7–13]. Nevertheless, further applications of aptamers in new research areas and clinical translation have been greatly hampered by their inherent limitations, including enzyme-mediated degradation and loss of targeting ability owing to their sensitivity to complex environments [14]. Therefore, the development of strategies to address these problems is one of the most attractive topics in FNA science [15–18].

In 2017, the Tan group pioneered the enzyme-mediated engineering of the monovalent aptamer to generate circular bivalent aptamers (CBApts) [19]. Compared with monovalent aptamers, CBApts exhibited markedly enhanced resistance to enzyme digestion and had considerably improved recognition ability. The CBApt concept was then extended to efficiently deliver functional proteins into target cancer cells [20,21], the design of novel aptamer-drug conjugates (ApDCs) for precise delivery of small molecule anticancer drugs [22,23], targeted T-cell immunotherapy for malignancies [24], as well as the diagnosis and treatment of tauopathy [25].

Although CBApts show great potential for use in the diagnosis and treatment of severe diseases, the current construction technology of CBApts faces several challenges. Successful engineering of CBApts requires the use of T4 ligase and the incorporation of 13 additional nucleosides at the end of one aptamer to form a double-stranded DNA with another aptamer with 13 complementary nucleosides [19]. Additionally, this method applies to only hairpin aptamers but not to other aptamers with the secondary structures of stem, loop, bugle, pseudoknot or G-quadruplex. These notable disadvantages may prohibit the divergent, large-scale preparation of CBApts, ultimately hindering their wider applications. Thus, the development of new synthetic methods for CBApt construction may provide a variety of recognition probes for investigations on accurate diagnosis and precise treatment of major diseases.

**Figure 1.**
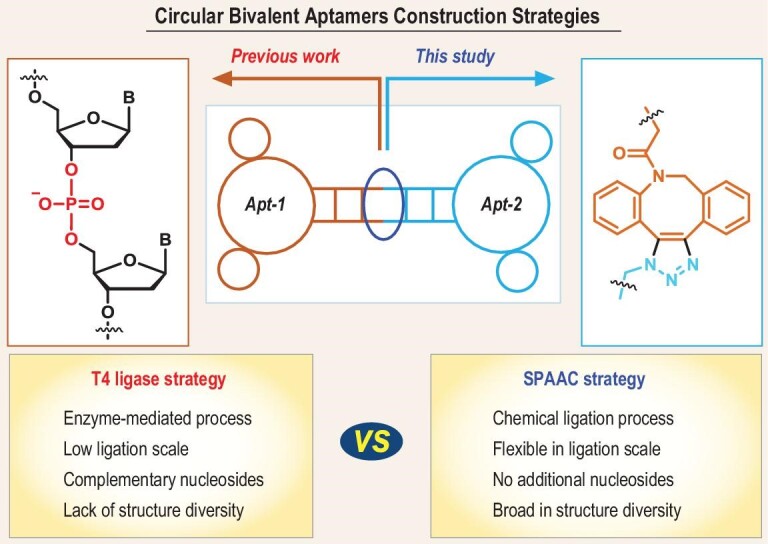
Brief structure of circular bivalent aptamers (CBApts) and the comparison of T4 ligase-based method and SPAAC-based method.

‘Click chemistry’—a term coined by Sharpless and co-workers—describes chemical transformations that are characterized by wide scope, high yields and negligible byproducts [26,27]. The utilization of these synthetic methodologies to generate biologically relevant constructs is of great interest, owing to their paramount importance in the facile production of molecular probes for pharmaceutical development and many other biomedical applications [28]. We envisioned that click chemistry may be a practical method to access CBApts because of its wild reaction conditions, ease of operation and diversity of reaction types. Herein, we describe the use of strain-promoted alkyne-azide cycloaddition (SPAAC) strategy in the divergent synthesis of CBApts, which showed considerably improved biological properties for biomedical applications (Fig. 1).

## RESULTS

### Optimization of reaction conditions

**Figure 2.**
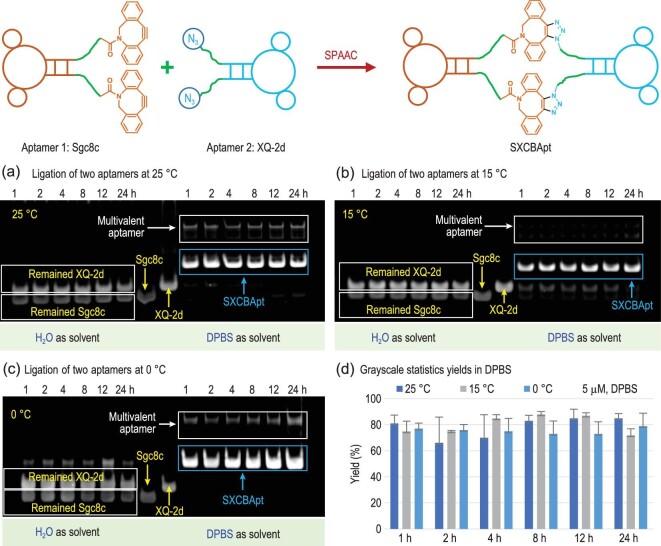
Optimization of the reaction conditions for Sgc8c∼XQ-2d CBApt (SXCBApt) construction. The reaction was carried out with Sgc8c and XQ-2d (5 μM) in DPBS buffer or pure water at different temperatures and times, respectively. Polyacrylamide gel electrophoresis (PAGE) analysis of the click reaction was conducted at (a) 25°C, (b) 15°C and (c) 0°C. Lane 1–Lane 6: 5′, 3′-diazide-labeled-sgc8c (N_3_-Sgc8c-N_3_) and 5′, 3′-diDBCO-labeled-XQ-2d (DBCO-XQ-2d-DBCO) reacted in pure water for 1, 2, 4, 8, 12 and 24 h, respectively; Lane 7: N_3_-Sgc8c-N_3_; Lane 8: DBCO-XQ-2d-DBCO; Lane 9–Lane 14: N_3_-Sgc8c-N_3_ and DBCO-XQ-2d-DBCO reacted in DPBS buffer for 1, 2, 4, 8, 12 and 24 h, respectively. (d) The ligation yields in DPBS of different reaction conditions (average yield of three runs; for details, see supporting information, Supplementary Table S2a).

Motivated by T4 ligase-enabled cyclization of the Sgc8c (targeting tyrosine-protein kinase 7, PTK7) [29] and XQ-2d (targeting transferrin receptor 1) [30] aptamers, and the high efficiency and mild reaction conditions of SPAAC, we hypothesized that the chemical cyclization of Sgc8c and XQ-2d could be achieved by incorporating an azide group at both the 5′- and 3′-termini of Sgc8c, as well as a dibenzocyclooctyne (DBCO) group at both the 5′- and 3′-termini of XQ-2d, resulting in the aimed Sgc8c∼XQ-2d CBApt (SXCBApt) (for DNA sequences, see supporting information, Supplementary Table S1). We first verified the effects of temperature, time and reaction media on the ligation efficiency of this SPAAC-promoted aptamer cyclization process using polyacrylamide gel electrophoresis (PAGE) analysis (Fig. 2; see mass spectrometry in Supplementary Fig. S1). Preparation of SXCBApt was carried out in Dulbecco's phosphate-buffered saline (DPBS) buffer under the following reaction conditions: 5 μM concentration of DNA, 1:1 molar ratio, 25°C and 1 h. This produced the desired product with ∼80% yield (Fig. 2a and d), which was improved to 85% by prolonging the reaction time to 12 h. Lowering the reaction temperature to 15°C improved the ligation yield to 88% (Fig. 2b and d), whereas slightly decreased ligation efficiency was observed at 0°C (Fig. 2c and d). In sharp contrast, little or no product was detected when the reaction was conducted in pure water under otherwise identical conditions, suggesting that the formation of a stable hairpin secondary structure is crucial for the successful cyclization of aptamers. Considering the much easier temperature control at 25°C vs. 15°C and the negligible difference in conjugation yield, we conducted subsequent reaction condition optimization at 25°C in DPBS buffer.

**Figure 3.**
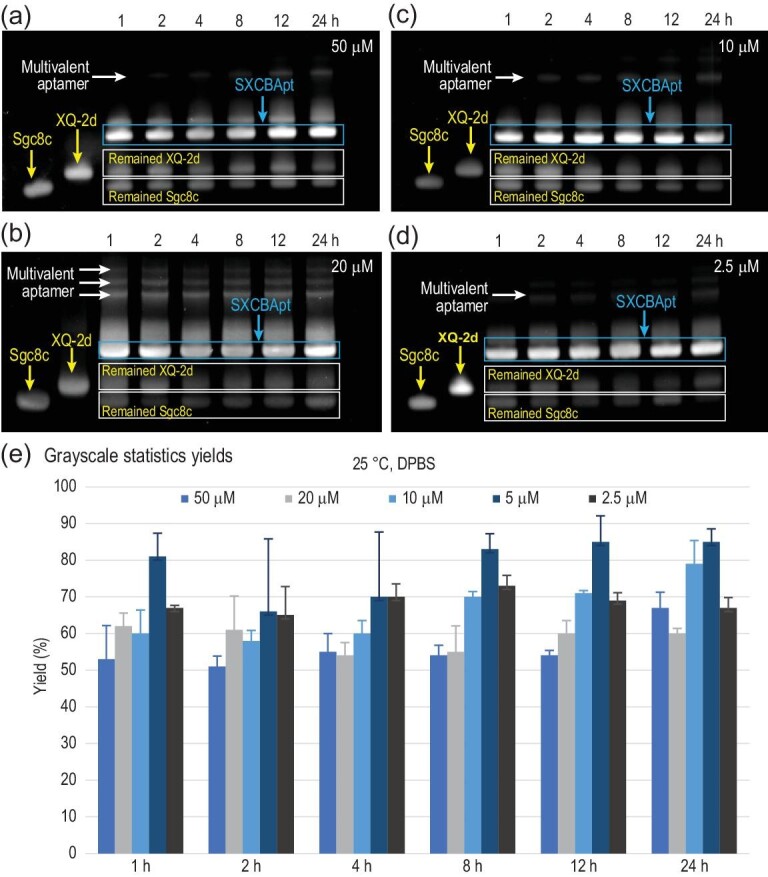
Reaction efficiency at different concentrations of Sgc8c and XQ-2d. For PAGE analysis of click reaction at 25°C in DPBS buffer, the reactant concentrations were (a) 50 μM, (b) 20 μM, (c) 10 μM and (d) 2.5 μM. Lane 1: N_3_-Sgc8c-N_3_; Lane 2: DBCO-XQ-2d-DBCO; Lane 3–Lane 8: N_3_-Sgc8C-N_3_ and DBCO-XQ-2d-DBCO reaction for 1, 2, 4, 8, 12 and 24 h, respectively. (e) The ligation yield (average yield of three runs; see Supplementary Table S2b) of different concentrations of Sgc8c and XQ-2d in DPBS, 25°C. The yield of 5 μM was calculated from Fig. 2a.

We next examined the effect of reactant concentration on SPAAC-promoted ligation in DPBS buffer at 25°C (Fig. 3). The ligation efficiency at the reactant concentrations of 50, 20, 10 and 2.5 μM was recorded from 1 to 24 h using PAGE and is summarized in Fig. 3e. As presented, 5 μM (grayscale statistics yields in Fig. 3e) was the best choice of DNA concentration, resulting in the highest yields regardless of the reaction time. SXCBApt was generated with an 85% yield after 12 h. Subsequently, we investigated the effect of the molar ratio of Sgc8c and XQ-2d on the reaction efficiency. When the molar ratio of Sgc8c and XQ-2d was 1:1, the reaction efficiency was the highest, but it decreased with the increase in the molar ratio of reactants (Supplementary Fig. S2). Based on the obtained results, we fixed the optimal reaction conditions at 5 μM concentration of azide/DBCO-modified aptamers in DPBS buffer, 1:1 molar ratio, 25°C and 12 h.

### The generality of the newly developed methodology

**Figure 4.**
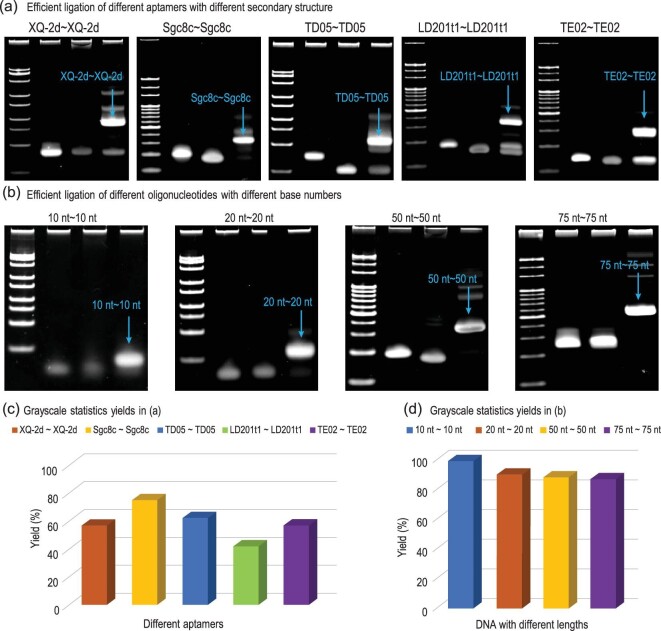
Reaction efficiency of different aptamers and DNA sequences at 25°C. (a) PAGE analysis of ligation efficiency of 5', 3'-azide-labeled and 5', 3'-DBCO-labeled aptamers at 25°C. (b) PAGE analysis of ligation efficiency 5', 3'-azide-labeled and 5', 3'-DBCO-labeled 10-base, 20-base, 50-base, 75-base DNA sequences at 25°C. (c) Ligation yields in (a). (d) Ligation yields in (b).

To determine whether this method could be used as a general strategy to construct CBApts, we performed the cyclization reaction with different aptamers in DPBS buffer at 25°C for 12 h (Fig. 4a). The targeted CBApts, including XQ-2d∼XQ-2d, Sgc8c∼Sgc8c, TD05∼TD05, LD201t1∼LD201t1 and TE02∼TE02, were generated with good yields of 57%, 75%, 62%, 45% and 57%, respectively (Fig. 4c), although the reaction conditions were not fully optimized. Considering the importance of cyclic DNAs in various biomedical areas, we further explored the utility of this methodology by examining the effect of the number of nucleosides on the ligation efficiency of various cyclic DNA entities with identical reaction conditions (Fig. 4b). Two short DNA sequences with 10 nucleosides each were coupled in an almost quantitative yield (99%) (Fig. 4d). The ligation of DNA sequences, each with 20 nucleosides, also produced the desired circular bivalent DNA with an excellent yield of 90%. Notably, increasing the number of nucleosides to 50 and 75 did not profoundly affect the ligation efficiency; the expected cyclic DNA sequences with 100 and 150 nucleosides were prepared with 88% and 87% yields, respectively.

After demonstrating the generality of the method, we designed and performed two groups of control experiments to verify the accurate creation of CBApts and cyclic DNAs. The products were purified and transferred to a vial to react with a 5′, 3′-diazide-labeled DNA sequence or a 5′, 3′-diDBCO-labeled DNA sequence, respectively. We believed that continued ligation would produce linear structures. On the contrary, the ligation process did not proceed with the cyclized products, as verified by analysing the gel images in Supplementary Fig. S3. This indicates the successful synthesis of CBApts and cyclic DNA architectures (simulation structure see Supplementary Fig. S4).

### Specific recognition ability of CBApts

**Figure 5.**
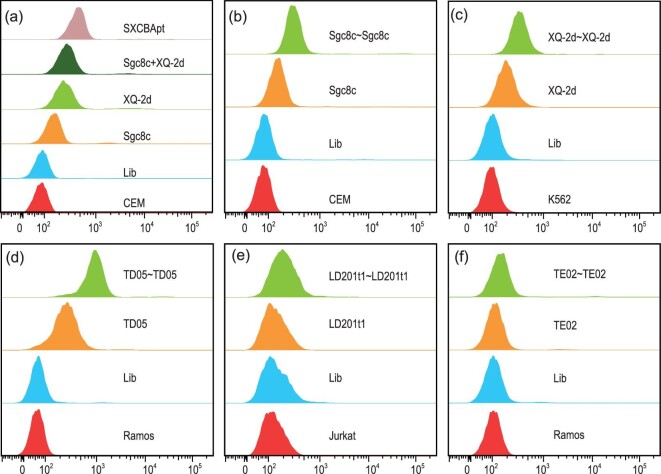
Comparison of the binding ability of CBApts with their corresponding monovalent aptamer at 250 nM and 4°C for 45 min using flow cytometry. (a) SXCBApt vs. XQ-2d and Sgc8c against CEM cells. (b) Sgc8c∼Sgc8c vs. Sgc8c against CEM cells. (c) XQ-2d∼XQ-2d vs. XQ-2d against K562 cells. (d) TD05∼TD05 vs. TD05 against Ramos cells. (e) LD201tl∼LD201tl vs. LD201tl against Jurkat cells. (f) TE02∼TE02 vs. TE02 against Ramos cells.

After establishing the method and demonstrating the correct structure of the ligated aptamers, we focused our attention on exploring their potential biomedical applications. Considering that specific recognition is one of the most important properties of aptamers, we tested the binding ability of CBApts. We expected that CBApts would exhibit better binding ability against cancer cells compared with monovalent aptamers, owing to their bis-ligand-receptor interaction models. To verify the binding ability of CBApts, we carried out flow cytometry experiments using Cy3-labeled CBApts. SXCBApt was used to investigate our hypothesis using CCRF-CEM cells (an acute lymphoblastic leukemia cell line) as the experimental target. As shown in Fig. 5a, both monovalent aptamers, Sgc8c and XQ-2d, specifically recognized the target cells. However, the results of the control group Sgc8c + XQ-2d indicated that the simple mix of these two monovalent aptamers did not enhance recognition ability. By contrast, SXCBApt displayed notably enhanced recognition ability, possibly explained by its unique dual-targeting model. Like SXCBApt, the bivalent Sgc8c∼Sgc8c aptamer also recognized the target CCRF-CEM cells more efficiently than the monovalent Sgc8c alone (Fig. 5b). Likewise, other CBApts, including XQ-2d∼XQ-2d (against K562 cells, a chronic myelogenous leukemia cell line), TD05∼TD05 and TE02∼TE02 (against Ramos cells, a human Caucasian Burkitt′s lymphoma cell line) and LD201t1∼LD201t1 (against Jurkat cells, a human T lymphocyte cell line) all exhibited considerably enhanced recognition ability compared with their corresponding monovalent aptamer (Fig. 5c–f; for Kd values, see Supplementary Fig. S5). These observations demonstrate that the chemical ligation strategy efficiently augmented the recognition ability of CBApts, which might be beneficial for biomedical applications.

### Internalization ability of CBApts

**Figure 6.**
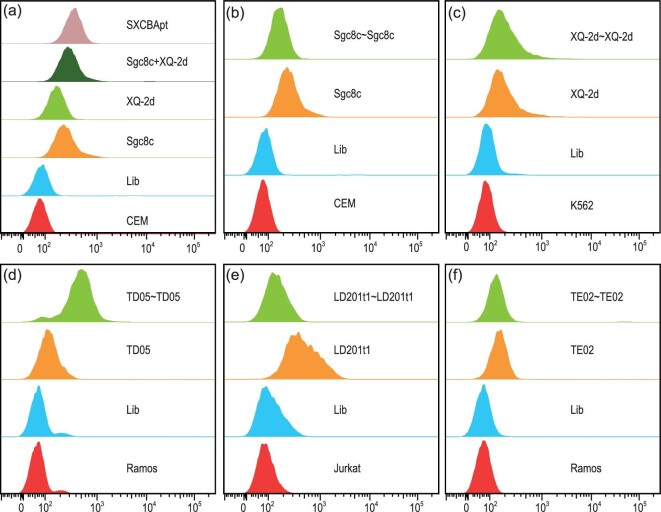
Comparison of the internalization ability of CBApts with their corresponding monovalent aptamer at 250 nM in RPMI-1640 media and 37°C for 2 h by flow cytometry. (a) SXCBApt vs. XQ-2d and Sgc8c against CEM cells. (b) Sgc8c∼Sgc8c vs. Sgc8c against CEM cells. (c) XQ-2d∼XQ-2d vs. XQ-2d against K562 cells. (d) TD05∼TD05 vs. TD05 against Ramos cells. (e) LD201tl∼LD201tl vs. LD201tl against Jurkat cells. (f) TE02∼TE02 vs. TE02 against Ramos cells.

It has been well established that aptamers possess excellent internalization ability, which could hold potential for various applications, e.g. aptamer-mediated targeted drug delivery using ApDC strategy [6]. The endocytosis ability of the newly developed Cy3-labeled CBApts was investigated and the outcomes of flow cytometry assays are summarized in Fig. 6. When CCRF-CEM was the target cell line, SXCBApt displayed better internalization ability than the monovalent aptamer components and the simple mixture of Sgc8c and XQ-2d (Fig. 6a), which might be explained by the fact that SXCBApts can recognize two different biomarkers on living cells to enhance aptamer–receptor interactions, leading to increased endocytosis ability [28]. However, the bivalent Sgc8c∼Sgc8c showed slightly lower internalization ability against CCRF-CEM cells even though its binding ability is superior to monovalent Sgc8c, which might be attributed to the fact that Sgc8c∼Sgc8c only interact with one kind of receptor on the cell membrane (Fig. 6b). This phenomenon is further confirmed by the XQ-2d∼XQ-2d and TE02∼TE02 aptamers, both of which entered their corresponding target cell lines as efficiently as the monovalent XQ-2d and TE02 aptamers (Fig. 6c and f). It is worth noting that while the monovalent TD05 loses its binding and internalization abilities at 37°C, the bivalent TD05∼TD05 aptamer exhibited markedly elevated internalization ability over TD05 alone with Ramos cells (Fig. 6d). This interesting result might be explained by the fixed secondary structure of TD05∼TD05 (Supplementary Fig. S4), which is beneficial for specific recognition. Unfortunately, extremely lower internalization ability was exhibited by LD201t1∼LD201t1 than by monovalent LD201t1 (Fig. 6e). Currently, we do not have an explanation for this observation. In addition, we also observed no obvious difference concerning the internalization ability of Sgc8∼Sgc8 before and after purification (Supplementary Fig. S6), indicating that purity is not an essential factor influencing the internalization ability of CBApt if the yield is high enough.

### Stability of CBApts in biological media

**Figure 7.**
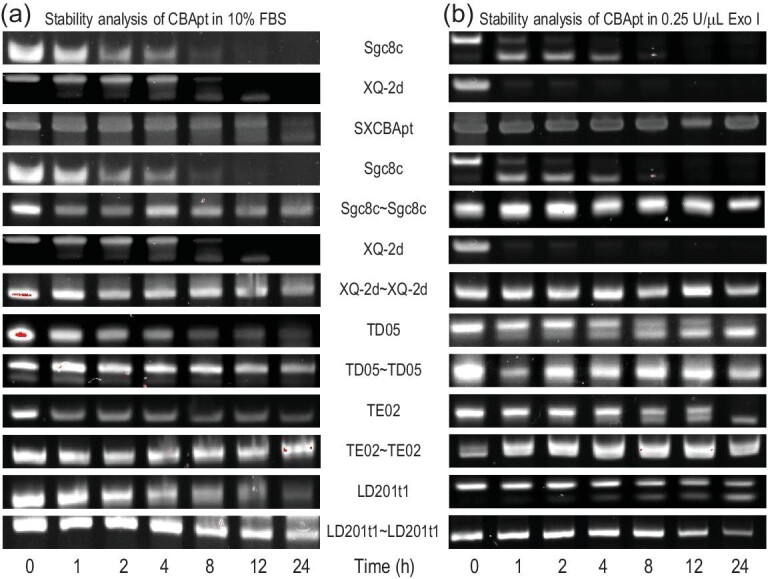
Stability analysis of CBApts and monovalent aptamers after treatment with 10% FBS and 0.25 U/μL Exo I. (a) Stability analysis of SXCBApt, Sgc8c∼Sgc8c, XQ-2d∼XQ-2d, TD05∼TD05, TE02∼TE02, LD201t1∼LD2021t1, Sgc8c, XQ-2d, TD05, TE02 and LD201t1 after incubation with 10% FBS for different periods, as determined using PAGE. (b) Stability analysis of Sgc8c∼XQ-2d, Sgc8c∼Sgc8c, XQ-2d∼XQ-2d, TD05∼TD05, TE02∼TE02, LD201t1∼LD2021t1, Sgc8c, XQ-2d, TD05, TE02 and LD201t1 after incubation with 0.25 U/μL Exo I for different periods, as determined using PAGE.

Since stability is an important characteristic of aptamers for biomedical applications, we explored the effect of cyclization on the stability of aptamers in biological media at a physiological temperature of 37°C. Monovalent aptamers were easily digested in 10% Fetal Bovine Serum (FBS) and their bands almost disappeared within 12 h (Fig. 7a). By contrast, the engineered CBApts retained their sequence integrity in 10% FBS even after 24 h of incubation. Considering that aptamers can also be digested by exonucleases, we compared the stability of monovalent aptamers and CBApts. The samples were incubated separately with 0.25 U/μL Exo I for different durations. Generally, monovalent aptamers were easily degraded after short incubation periods (Fig. 7b), whereas CBApt exhibited excellent exonuclease resistance even after 24 h of incubation with 0.25 U/μL Exo I. Therefore, aptamer cyclization considerably increased the stability of CBApts in biological media.

### Enhanced cell–cell interaction

**Figure 8.**
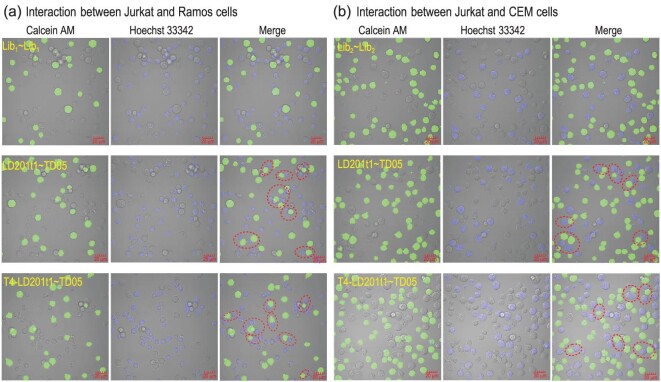
Circular bispecific aptamer-mediated cell–cell interaction. Representative confocal micrographs showing junctional cell–cell complexes after treatment with (a) Lib_1_∼Lib_1_, LD201t1∼TD05 and T4-LD201t1∼TD05 for regulating Jurkat and Ramos cells; (b) Lib_2_∼Lib_2_, LD201t1∼Sgc8c and T4-LD201t1∼Sgc8c for regulating Jurkat and CEM cells. Jurkat cells were stained with calcein AM, whereas Ramos cells and CCRF-CEM cells were unstained with Hoechst 33342. Scale bars represent 20 μm.

To explore the potential biomedical application of CBApts, we designed a CBApts-mediated ‘recognition-then-interaction’ strategy for regulating cell behaviors. We envisioned that CBApt could simultaneously bind to the target receptors on two different types of cells, thereby allowing them to adhere to each other to form junctional cell–cell complexes, thus changing the behavior of cells. To verify this hypothesis, the LD201t1 aptamer that can specifically recognize CD62L (highly expressed on the surfaces of T-cell membranes) and tumor cell-targeting aptamers (sgc8c, a CCRF-CEM targeting aptamer; TD05, a Ramos targeting aptamer) were chosen to construct the designed CBApts. Next, we evaluated the cell–cell linkage efficiency of Jurkat and Ramos cells or Jurkat and CCRF-CEM cells. First, calcein AM-pre-labeled Jurkat cells were mixed with Ramos or CCRF-CEM cells in a 1:5 ratio and then incubated with the CBApts, LD201t1∼TD05, T4-LD201t1∼TD05, LD201t1∼Sgc8c, T4-LD201t1∼Sgc8c or Lib∼Lib (library control groups) in binding buffer at 4°C for 1 h. Cell–cell interactions were monitored using confocal imaging, which indicated the formation of junctional cell–cell complexes when Jurkat and Ramos cells were incubated with LD201t1∼TD05 and T4-LD201t1∼TD05 (Fig. 8a). In contrast, no cell–cell interactions were detected when Jurkat and Ramos cells were incubated with Lib_1_∼Lib_1_. Notably, similar cell–cell interaction was observed when CCRF-CEM and Jurkat cells were incubated with LD201t1∼Sgc8c and T4-LD201t1∼Sgc8c under otherwise identical conditions (Fig. 8b). Thereafter, Jurkat cells were pre-labeled with calcein AM and Ramos and CCRF-CEM cells were pre-labeled with Hoechst dye for flow cytometry analysis (Supplementary Fig. S7), to further identify the observed cell–cell interaction using the double-stained cell population identification technique. The results were consistent with confocal microscopy observations, indicating that the CBApts engineered by chemical ligation significantly enhanced cell–cell interaction, which may provide a new method for regulating cell behaviors.

### Efficient *in vivo* (mice) recognition ability of CBApts

**Figure 9.**
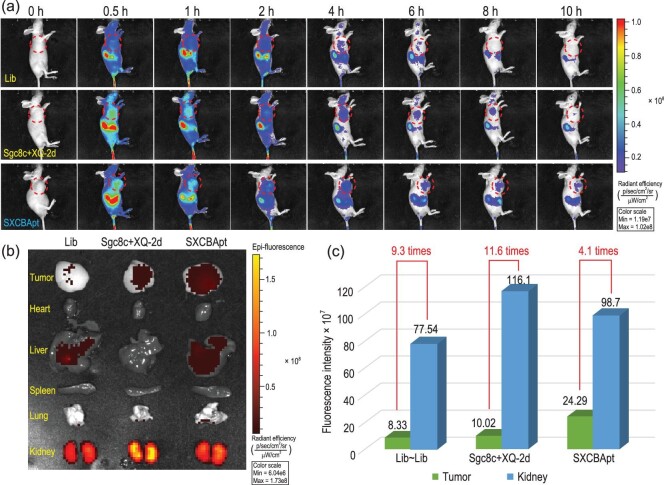
(a) *In vivo* fluorescence imaging of CCRF-CEM tumor-bearing mice after Cy5-labeled Lib, Cy5-labeled Sgc8c + XQ-2d and Cy5-labeled SXCBApt were injected through the tail vein. (b) Distribution of Cy5-labeled Lib, Cy5-labeled Sgc8c + XQ-2d and Cy5-labeled SXCBApt in the tumor and major viscera (heart, liver, spleen, lung, kidney) at the organ level 10 h post intravenous injection. (c) Comparing the fluorescence intensity of Cy5-labeled Lib, Cy5-labeled Sgc8c + XQ-2d and Cy5-labeled SXCBApt in the tumor, Cy5-labeled Sgc8c + XQ-2d and Cy5-labeled library DNA sequence in the kidney.

Targeted *in vivo* imaging experiments were carried out to test the *in vivo* application potential of CBApts using CCRF-CEM tumor-bearing mouse models. We prepared Cy5-labeled CBApt (Cy5-SXCBApt) using Cy5-labeled Sgc8 and XQ-2d. Clear fluorescence signals were detected at the tumor sites within the Cy5-SXCBApt and Cy5-labeled Sgc8c + XQ-2d groups at 0.5 h (Fig. 9a), demonstrating superior enrichment ability of aptamers over a library DNA sequence (Lib). The Cy5 signals in these two groups could still be detected at 10 h post injection, whereas the fluorescence signal of Cy5-labeled Lib rapidly diminished and almost completely disappeared at 8 h post injection. The experimental mice were then sacrificed to collect major organs to record the fluorescence images. As presented in Fig. 9b, Lib, Sgc8c + XQ-2d and SXCBApt groups accumulated mostly in kidneys. However, their tumor tissue gathering ability was quite different. Superiorly higher organ selectivity ability was exhibited by the SXCBApt group. The ratio of the amount of aptamer distributed in the kidney to that distributed at the tumor site for the SXCBApt group was 4.1 but was 9.3 and 11.6 for Lib and Sgc8c + XQ-2d, respectively (Fig. 9c). To see whether there is any difference in the targeting ability of CBApts constructed using the T4 enzymatic method and chemical coupling in tumor tissues, we compared the fluorescence intensity of SXCBApt and T4-SXCBApt in tumor tissues using the nude mouse *in vivo* imaging technology and found that the SXCBApt group exhibited superior targeting and accumulation abilities over the T4-SXCBApt group demonstrated by the fluorescence signal at the time point of 6 h for SXCBApt group but not T4-SXCBApt group (Supplementary Fig. S8). This experimental result indicates that the construction method of CBApts does have a certain level of influence on their recognition ability towards tumor tissue.

## DISCUSSION

To effectively improve the biomedical properties of FNAs and their application potential in the field of biomedicine, we developed a SPAAC-based chemical ligation strategy using nucleic acid aptamers as starting materials for the engineering of CBApts. After detailed optimization of the reaction parameters, including temperature, reaction medium, concentration and time, we obtained the optimal reaction conditions of this technology: the concentration of N_3_- and DBCO-modified aptamers is 5 μM, the molar ratio of these aptamers is 1:1 and the reaction temperature in DPBS buffer is 25°C for 12 h. After establishing the optimal reaction conditions, the versatility of the method was investigated. It was found that the method could be applied to different aptamers and single-stranded nucleic acids with different lengths and the target circularization can be obtained with a moderate to excellent yield. These results indicated that the length and secondary structure of the monovalent aptamers or single-stranded nucleic acids have significant impacts on the coupling efficiency. This is because the conformation of nucleic acids in the solution changes dynamically, causing a differential collision of the functional groups.

We compared the biological properties of CBApts with monovalent aptamers and found that the stability of bivalent aptamers in 10% FBS and 0.25 U/μL Exo I was much higher than that of monovalent aptamers. This may be related to the rigid structure of CBApt, lowering the recognition and degradation by nuclease. In addition, in terms of its binding ability to target cells, CBApt has different degrees of improvement as compared to the monovalent aptamers. The reason for this difference may be attributed to the different binding modes (two different ligands target two different receptors vs. two identical ligands target the same receptor) or the difference in the expression of receptors on the cell membrane surface. Although the binding capacity of the bivalent nucleic acid aptamers is greatly improved compared with the monovalent aptamer, their cell internalization ability shows significant differences—some are improved (such as SXCBApt and TD05∼TD05), some are reduced (Sgc8c∼Sgc8c, LD201t1∼LD201t1) and some are at the same level (XQ-2d∼XQ-2d, TE02∼TE02), which may be related to the different internalization pathways of the receptor.

To better examine the potential application of CBApt in the field of biomedicine, we designed a ‘recognition-then-interaction’ strategy to regulate cell interactions. Benefitting from the specific recognition ability and high-affinity binding function of the aptamer to the target, two kinds of cells that could not interact with each other were attracted and approached to each other, which provides new technical support for the application of aptamers for the regulation of cell behavior. On this basis, we further explore the possibility of applying CBApt *in vivo*. CBApt is much better than simply using a mixture of two aptamers to selectively accumulate in tumor tissues and the retention time is also greatly extended. We also compared CBApt obtained using the methodology described herein with the CBApt obtained using the T4 ligase method and found that SXCBApt exhibited a superior ability to recognize tumors and accumulate and retain in tumor tissues than T4-SXCBApt, indicating that the construction methods indeed have effects on biological properties of the bivalent nucleic acid aptamers.

## CONCLUSIONS

To promote further utilization of aptamers in biomedical research, we developed a convenient yet efficient chemical ligation strategy to assemble multiple types of CBApts with significantly improved nuclease resistance ability and binding affinity. Unlike the ligase-mediated cyclization technique, this newly developed approach does not require the incorporation of complementary nucleosides at the end of each aptamer to form a double-stranded DNA. This method can also be utilized to construct cyclized DNA architectures using non-sticky end reactants and small hairpin DNA molecules. The application studies demonstrated that SXCBApt selectively recognized tumor tissue and was retained at the tumor site for a longer time. Moreover, the CBApts might be utilized to design a ‘recognition-then-interaction’ strategy for regulating cell behaviors. The considerable advantages of this technique suggest that it could be applied extensively in a wide range of biomedical areas in the future.

## METHODS

### General procedure for the construction of CBApts

5′, 3′-diazide-labeled oligonucleotides (ODNs) and 5′, 3′-diDBCO-labeled oligonucleotides (ODNs) with the molar ratio of 1:1 were mixed in DPBS (the final concentration of each oligonucleotide was 5 μM); the mixture was then allowed to react at 25°C for 12 h. The yield was assessed by using Image Lab software to calculate the gray value of the PAGE glue; the gray value of the targeted product was divided by the overall gray value of all substances in the reaction media.

### General procedure for native PAGE

To characterize the correct construction of CBApts, each sample (10 μL, 2 μM) was mixed with 6 × loading buffer (2 μL). Electrophoresis was performed in 1 × Tris-acetic acid EDTA buffer (pH = 8.34) at 110 V for 45 min. After that, the polyacrylamide gel was stained with GelRed for 15 min and then imaged on a Bio-Rad molecular imager with UV light.

### General procedure for *in vivo* fluorescence imaging

Healthy female BALB/c mice were purchased from Hunan SJA Laboratory Animal Co. Ltd. Animal care and the related handling procedures were carried out according to the guidelines of the Institutional Animal Care and Use Committee of Hunan University and also by guidelines of the Regional Ethics Committee for Animal Experiments. All animal-related experimental procedures were approved by the Animal Care and Use Committee of Hunan University (SYXK 2018-0006). The animals were allowed free access to sterile water and food. Four- to 6-week-old BALB/c nude mice received a subcutaneous injection of 1 × 10^7^ CCRF-CEM cells in the right axilla. Tumors were then allowed to grow over 21–28 days until the tumor volume reached ∼300 mm^3^. Tumor-bearing BALB/c nude mice were anesthetized to be motionless with both tranquilizer and anesthetic before 5 nmol of Cy5-labeled SXCBApt, Sgc8 + XQ-2d or library was injected intravenously via the tail vein. At the designated time points, mice were anesthetized using an isoflurane vaporizer and fluorescence images of live mice were collected using an IVIS Lumina II *in vivo* imaging system (Caliper LiveScience, USA). Mice were injected with the Cy5-labeled SXCBApt, Sgc8 + XQ-2d or library. After 10 h, their main organs (brain, heart, liver, spleen, lung and kidney) were carefully collected for *ex vivo* imaging using an IVIS Lumina II *in vivo* imaging system.

## Supplementary Material

nwac107_Supplemental_File
