# Supplementary material for: A versatile strategy for convenient circular bivalent functional nucleic acids construction
Source: Natl Sci Rev. 2022 Jun 7;10(2):nwac107. doi: 10.1093/nsr/nwac107 (PMC10029841; doi:10.1093/nsr/nwac107)
Supplement: nwac107_Supplemental_File [file nwac107_supplemental_file.docx]

Supplementary Data

For

A Versatile Strategy for Convenient Circular Bivalent Functional Nucleic Acids Construction

Xiao-Jing Zhang^a^, Zhuo Zhao^a^, Xia Wang^a^, Min-Hui Su^a^, Lili Ai^a^, Yingying Li^a^, Quan Yuan^a^, Xue-Qiang Wang^a,^* , Weihong Tan^a, b, c^

^a^ Molecular Science and Biomedicine Laboratory (MBL), State Key Laboratory of Chemo/Biosensing and Chemometrics, College of Chemistry and Chemical Engineering, College of Biology, Aptamer Engineering Center of Hunan Province, Hunan University, Changsha 410082, China

^b^ Institute of Molecular Medicine (IMM), Renji Hospital, Shanghai Jiao Tong University School of Medicine, and College of Chemistry and Chemical Engineering, Shanghai Jiao Tong University, Shanghai 200240, China

^c^ The Cancer Hospital of the University of Chinese Academy of Sciences (Zhejiang Cancer Hospital), Hangzhou Institute of Medicine (HIM), Chinese Academy of Sciences, Hangzhou, 310022, China

*Corresponding author. Email: wangxq@hnu.edu.cn

**Reagents and materials.**

All DNA oligonucleotides were purchased from Sangon Biotech Co., Ltd. (Shanghai, China). 2-amino-2-(hydroxymethyl)-1,3-propanediol (Tris, 99.9%) were purchased from Beyotime Co. (Jiangsu, China). Ethylenediaminetetraacetic acid disodium salt dihydrate (EDTA, AR, 98%) was purchased from Aladdin Industrial Co. (Shanghai, China), 30% acrylamide was purchased from Servicebio (Wuhan, China), ammonium persulfate (APS, 98%) was purchased from Sinopharm Chemical Reagent Co., Ltd (Shanghai, China), GelRed was purchased from US Everbright® Inc. (Suzhou, China), Exonuclease I (Exo I) with 10× Exo I buffer (670 mM Glycine-KOH, pH 9.5, containing 10 mM DTT and 67 mM MgCl_2_) were purchased from Takara Biotechnology Co. (Dalian, China). Calcein AM and Hoechst Dye were purchased from Yeasen Biotechnology Co., Ltd. (Shanghai, China).

**Cell lines and cell culture.**

All cells used in this experiment, including CCRF-CEM, Ramos, K562, and Jurkat cells were purchased from ATCC. All cells were cultured in RPMI medium 1640 (Gibco) supplemented with 10% (v/v) fetal bovine serum (FBS, Gibco) and 1% penicillin-streptomycin solution (PS) at 37 °C in a 5% CO_2_ atmosphere. Dulbecco’s Phosphate Buffered Saline (DPBS, Gibco) was used to wash cells.

**Washing buffer and binding buffer.**

Washing buffer was prepared with DPBS supplemented with 4.5 g/L of glucose and 5 mM of MgCl_2_. Binding buffer was prepared with DPBS supplemented with 4.5 g/L of glucose, 5 mM of MgCl_2_, 0.1 mg/mL of yeast tRNA, and 1 mg/mL of BSA.

**Mathods.**

**General procedure for stability analysis of aptamers in exonuclease solution and serum**. Briefly, 2 μM Cy3-labeled monovalent aptamers, and CBApts were incubated with 0.25 U/μl exonuclease I (Exo I) or RPMI 1640 with 10% fetal bovine serum (FBS) at 37 °C. At designated time points (0, 1, 2, 4, 8, 12, 24 h), samples were heated at 95 °C for 5 min to denature the enzyme and subsequently stored at -20 °C until all samples were collected. Samples were then thawed on ice for electrophoresis assays.

**General procedure for flow cytometry assays**. a) For the binding ability experiment, 4×10^5^ cells (CCRF-CEM, Ramos cells, K562 cells, or Jurkat cells) were washed with washing buffer via centrifugation at 1000 r/min and then incubated with Cy3-labeled CBApt, ssDNA, or library in 200 μL of binding buffer at 4 °C for 45 min for binding assay, or 200 μL of RPMI 1640 with 10% FBS at 37 °C for 2 h for endocytosis assay. After washing twice with washing buffer (4 °C for binding or 37 °C for internalization), the samples were collected and then resuspended in 200 μL of washing buffer for flow cytometry. Flow cytometry was performed using a BD FACSVerse™ flow cytometer for cell detection.

b) For the junctional cell-cell efficiency experiment, calcein AM and Hoechst dye were pre-dissolved in DMSO. The cells were washed twice in DPBS buffer and then stained. Jurkat cells were labeled with calcein AM, meanwhile, Ramos cells and CCRF-CEM cells were labeled with Hoechst. Next, stained cells were washed twice with cold DPBS buffer and suspended in binding buffer at 4 °C. Thereafter, 3×10^5^ calcein AM-labeled Jurkat cells were incubated with 1 μM CBApt (LD201t1~TD05, and LD201t1~Sgc8c) or 1 μM nonspecific sequences (Lib~lib) in the binding buffer for 30 min at 4 °C. Subsequently, the same number of cancer cells (e.g., Ramos or CCRF-CEM cells) were added to the Jurkat cells solution, and then, the cell mixtures were further incubated at 4 °C for another 1 h, the co-stained cells were detected using a BD FACSVerse™ flow cytometer.

**General procedure for** **confocal laser scanning microscopy imaging**. Jurkat cells were washed twice with DPBS buffer and then incubated with calcein AM for 15 min at 37 °C. After washing with cold DPBS buffer, they were mixed with unstained Ramos cells or CEM cells in a ratio of 1:5 and then treated with 1 μM CBApt (LD201t1~TD05 and LD201t1~Sgc8c) or 1 μM nonspecific sequences (Lib~lib) in the binding buffer at 4 °C for 1 h. Then the images of junctional cell-cell complexes were recorded by confocal microscopy.

**Table S1:** Oligonucleotide sequences used in this study.

| Name | Strand components (5’-3’) |
| --- | --- |
| N_3_-Sgc8c-N_3_ | N_3_-ATC TAA CTG CTG CGC CGC CGG GAA AAT ACT GTA CGG TTA GA-N_3_ |
| DBCO-Sgc8c-DBCO | DBCO-ATC TAA CTG CTG CGC CGC CGG GAA AAT ACT GTA CGG TTA GA-DBCO |
| N_3_-XQ-2d-N_3_ | N_3_-ACT CAT AGG GTT AGG GGC TGC TGG CCA GAT ACT CAG ATG GTA GGG TTA CTA TGA GC-N_3_ |
| DBCO-XQ-2d-DBCO | DBCO-ACT CAT AGG GTT AGG GGC TGC TGG CCA GAT ACT CAG ATG GTA GGG TTA CTA TGA GC-DBCO |
| N_3_-TD05-N_3_ | N_3_-AGG AGG ATA GTT CGG TGG CTG TTC AGG GTC TCC TCC T-N_3_ |
| DBCO-TD05-DBCO | DBCO-AGG AGG ATA GTT CGG TGG CTG TTC AGG GTC TCC TCC T-DBCO |
| N_3_-TE02-N_3_ | N_3_-TAG GCA GTG GTT TGA CGT CCG CAT GTT GGG AAT AGC CAC GCC T-N_3_ |
| DBCO-TE02-DBCO | DBCO-TAG GCA GTG GTT TGA CGT CCG CAT GTT GGG AAT AGC CAC GCC T-DBCO |
| N_3_-LD201t1-N_3_ | N_3_-TAG CCA AGG TAA CCA GTA CAA GGT GCT AAA CGT AAT GGC TTC GGC TTA C-N_3_ |
| DBCO-LD201t1-DBCO | DBCO-TAG CCA AGG TAA CCA GTA CAA GGT GCT AAA CGT AAT GGC TTC GGC TTA C-DBCO |
| N_3_-10 nt-N_3_ | N_3_-CGT TAG ACG A-N_3_ |
| DBCO-10 nt-DBCO | DBCO-CGT TAG ACG A-DBCO |
| N_3_-20 nt-N_3_ | N_3_-GGT CTC CAT GTG TAG AAG AC-N_3_ |
| DBCO-20 nt-DBCO | DBCO-GGT CTC CAT GTG TAG AAG AC-DBCO |
| N_3_-50 nt-N_3_ | N_3_-CAT GCA TCT AAC TGC TGC GCC GCC GGG AAA ATA CTG TAC GGT TAG ATG CA-N_3_ |
| DBCO-50 nt-DBCO | DBCO-CAT GCA TCT AAC TGC TGC GCC GCC GGG AAA ATA CTG TAC GGT TAG ATG CA-N_3_ |
| N_3_-75 nt-N_3_ | N_3_-ATG CAT AGC ATG AGT TCA GTC GTA GTA ATG GTC CTT AGT CTT GCT TGG TGT TTC CTG ATG GCT CTC ATG CTA TGC-N_3_ |
| DBCO-75 nt-DBCO | DBCO-ATG CAT AGC ATG AGT TCA GTC GTA GTA ATG GTC CTT AGT CTT GCT TGG TGT TTC CTG ATG GCT CTC ATG CTA TGC-DBCO |
| Lib | ATC TAA CTG ATT ATT ATT ATT ATT ATT ATT ATT CGG TTA GA |

**Table S2**. The yield of three runs for preparing CBApts.


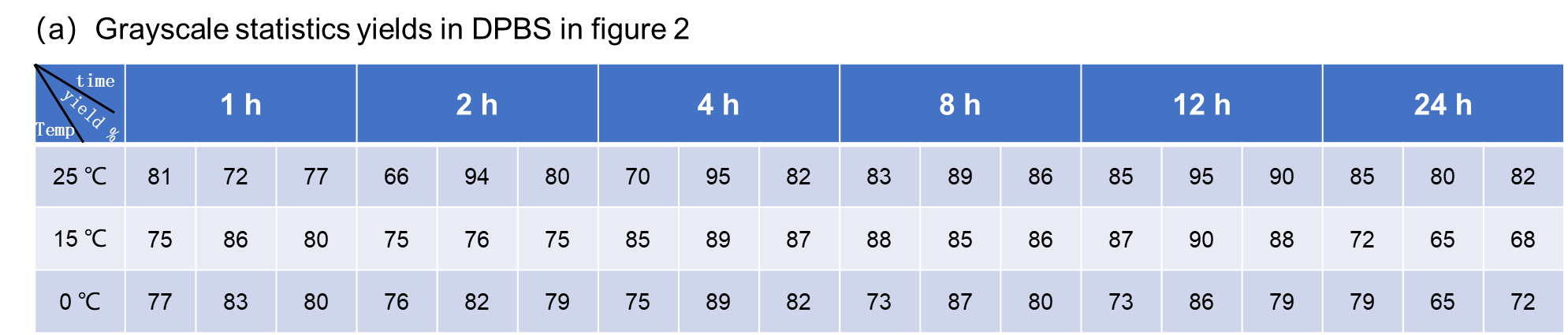


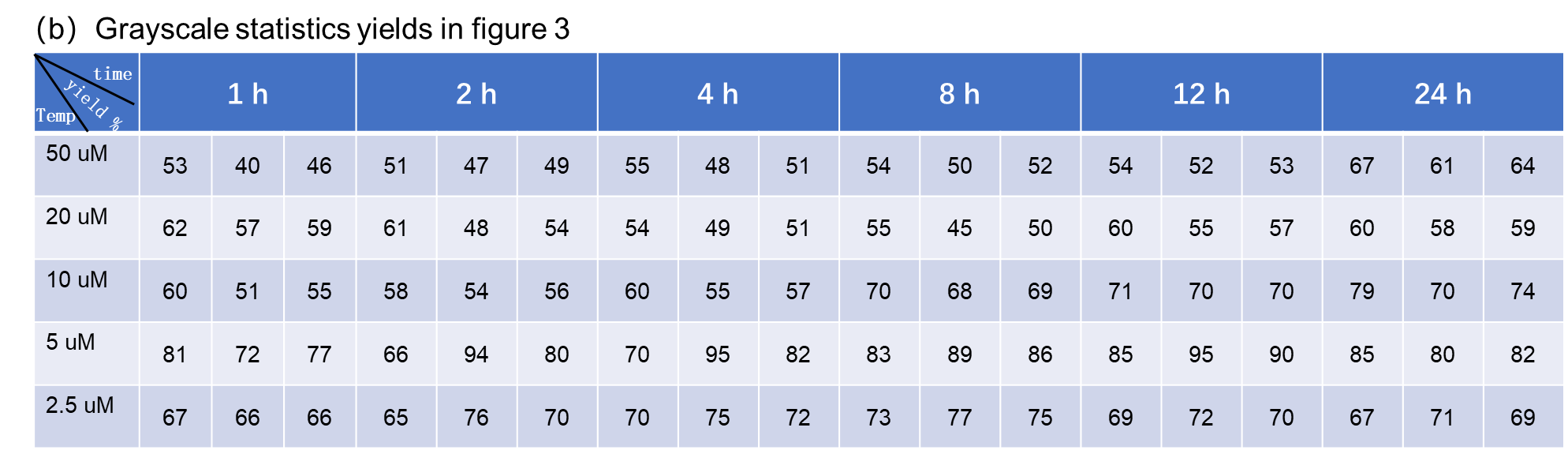


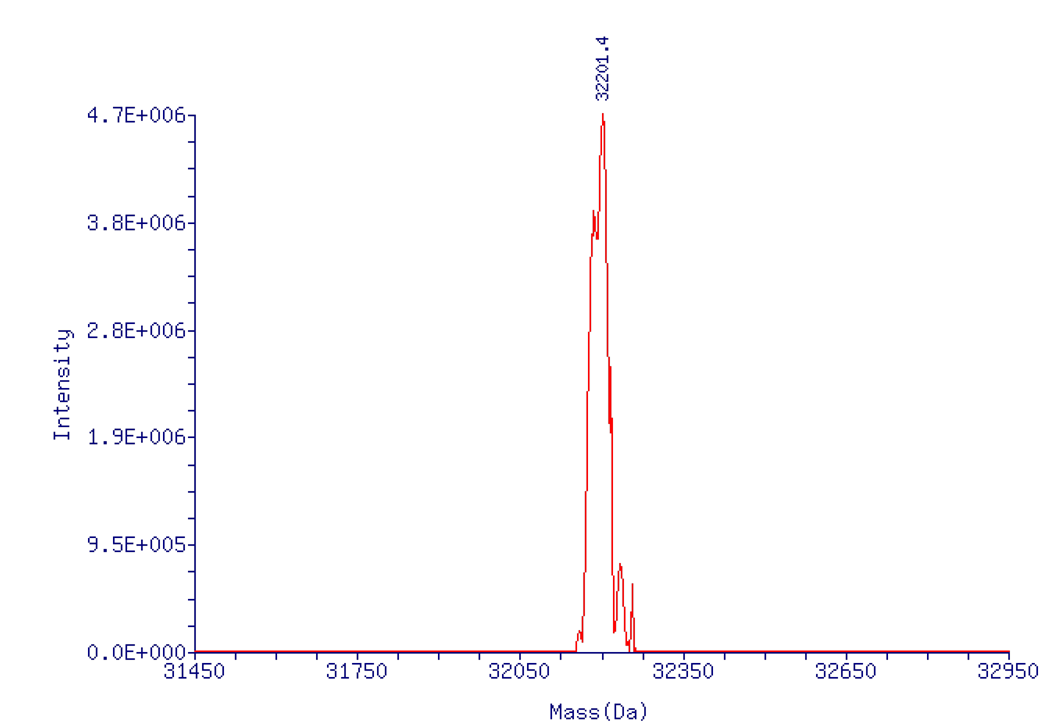


**Figure S1.** MS measurements of the SXCBApt.


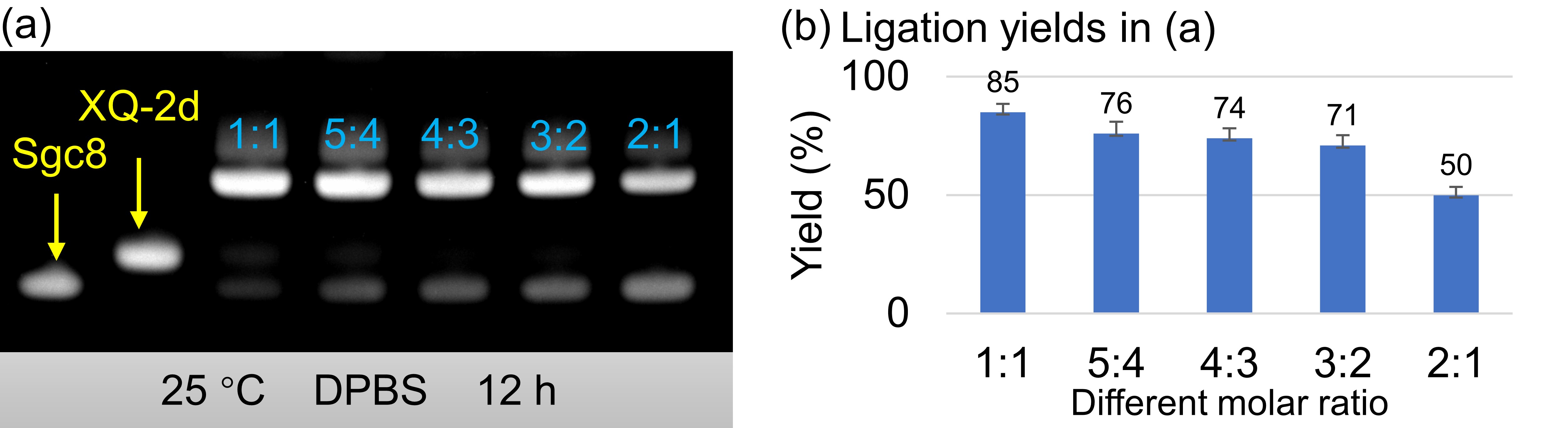


**Figure S2.** Effect of molar ratio of reactants on reaction efficiency. (a) Polyacrylamide gel electrophoresis was used to analyze the reaction efficiency of SXCBApt when the molar ratios of Sgc8c and XQ-2d were 1:1, 5:4, 4:3, 3:2, and 2:1, respectively, in DPBS buffer at 25 °C for 12 h. (b) Grayscale statistics yields of different molar ratios of Sgc8c and XQ-2d in DPBS buffer at 25 °C for 12 h.


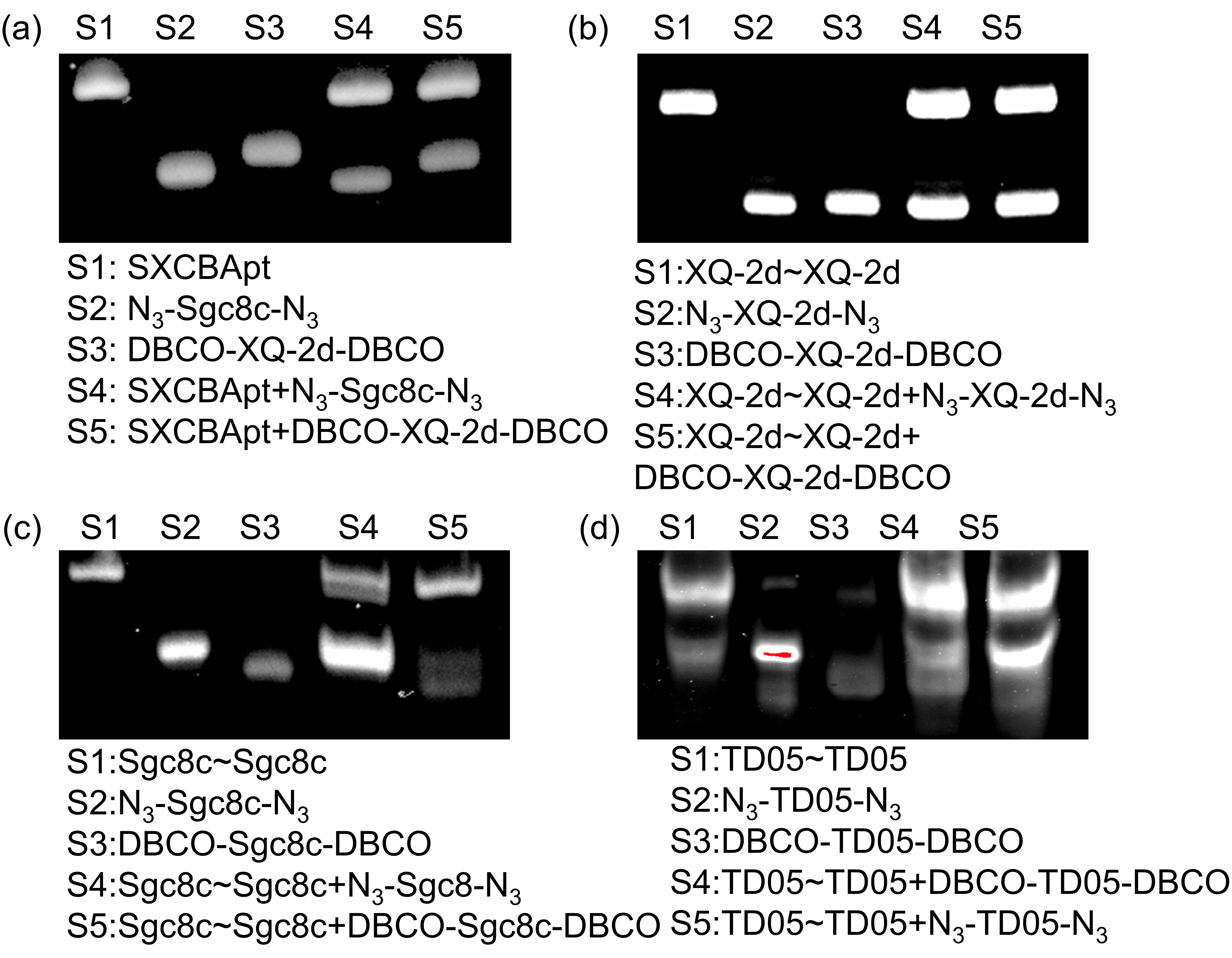


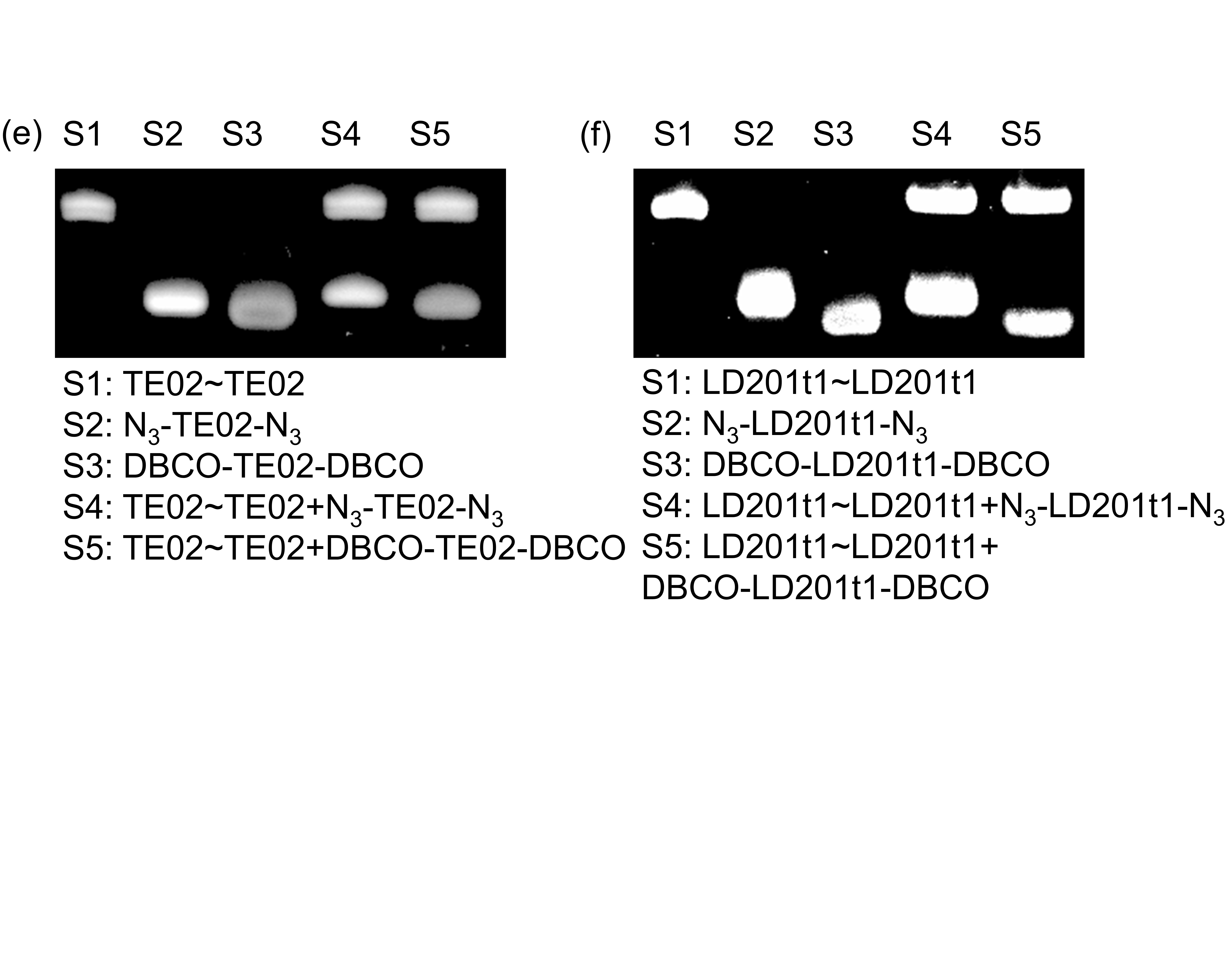


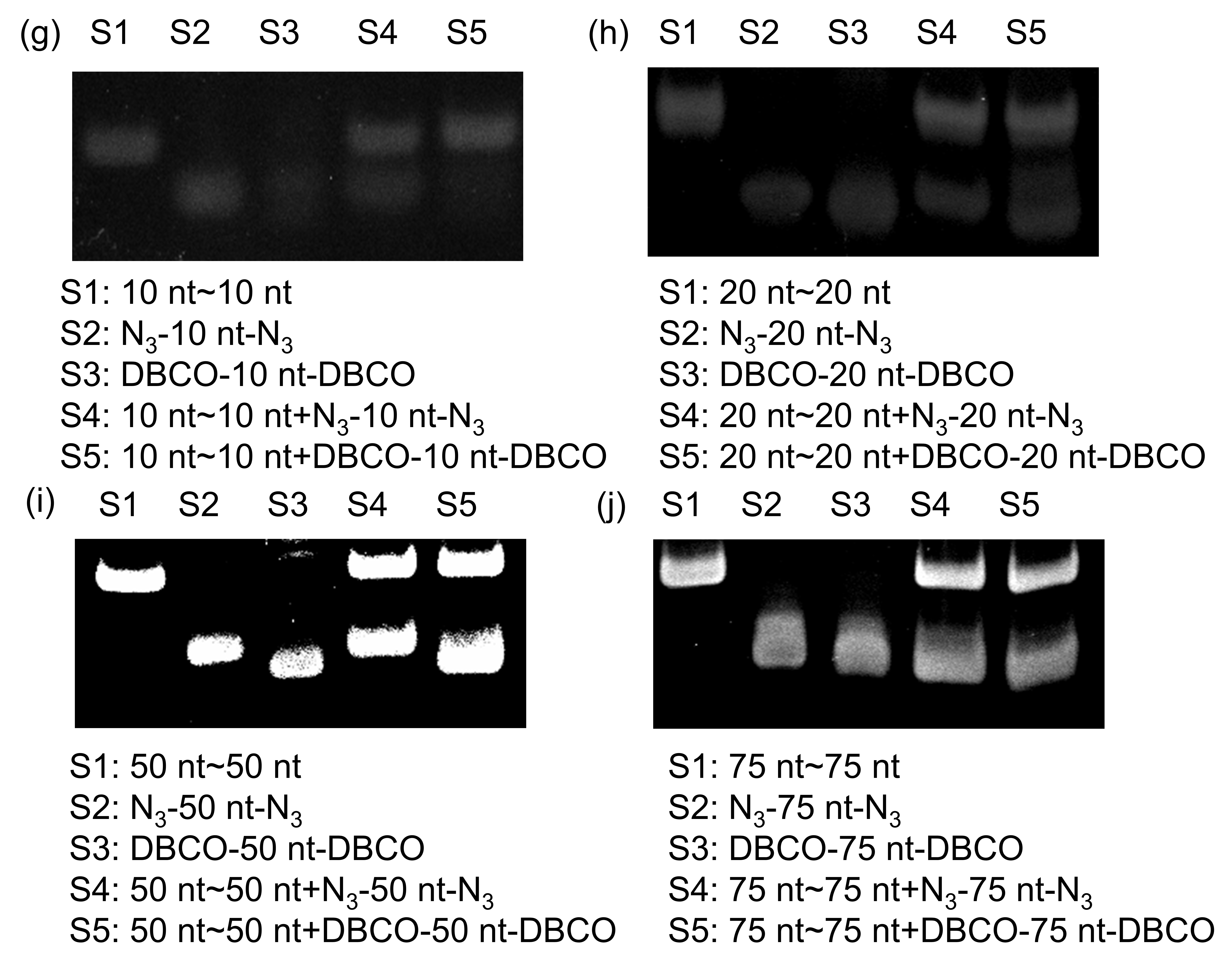


**Figure S3.** The demonstration of circular bivalent DNA structures. Polyacrylamide gel electrophoresis analysis of the circular bivalent structure reacted with 5’, 3’-diazide-labeled DNA sequence or 5’, 3’-diDBCO-labeled DNA sequence, respectively. The reaction conditions are the same as the conditions used to construct the circular bivalent structure.


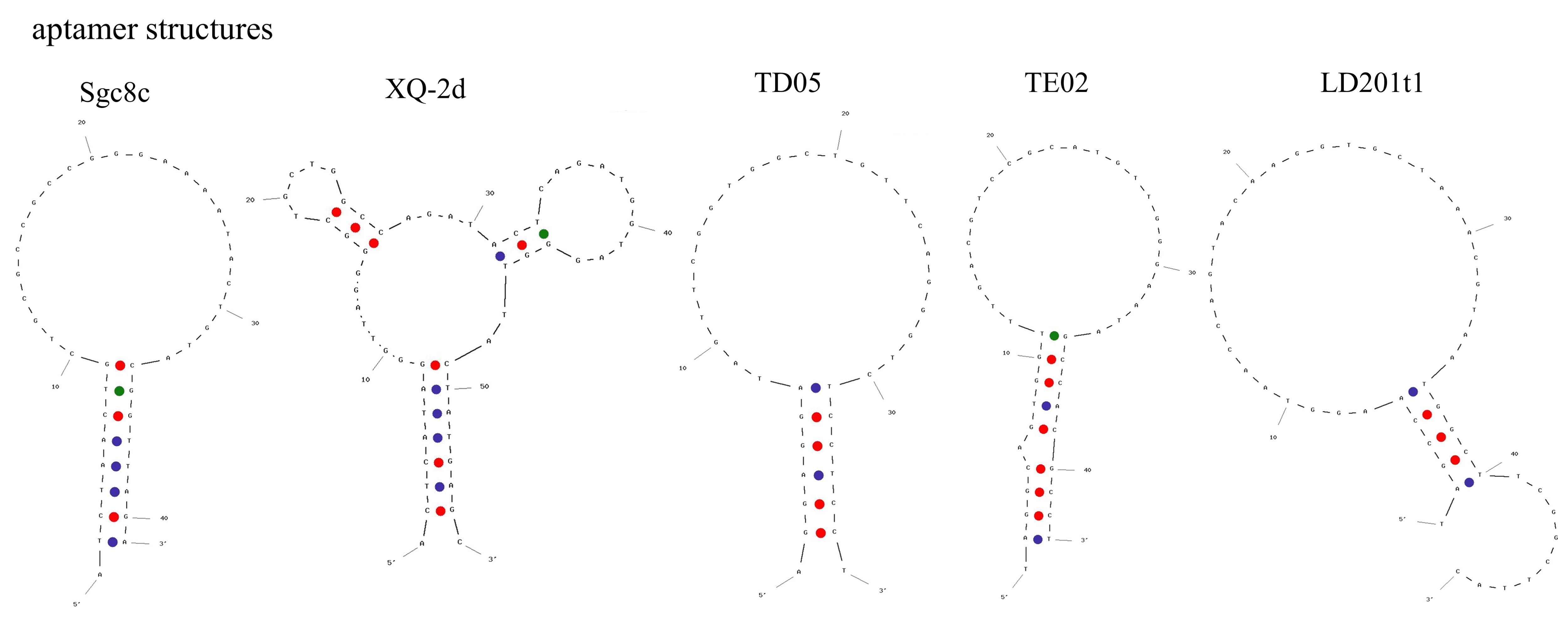

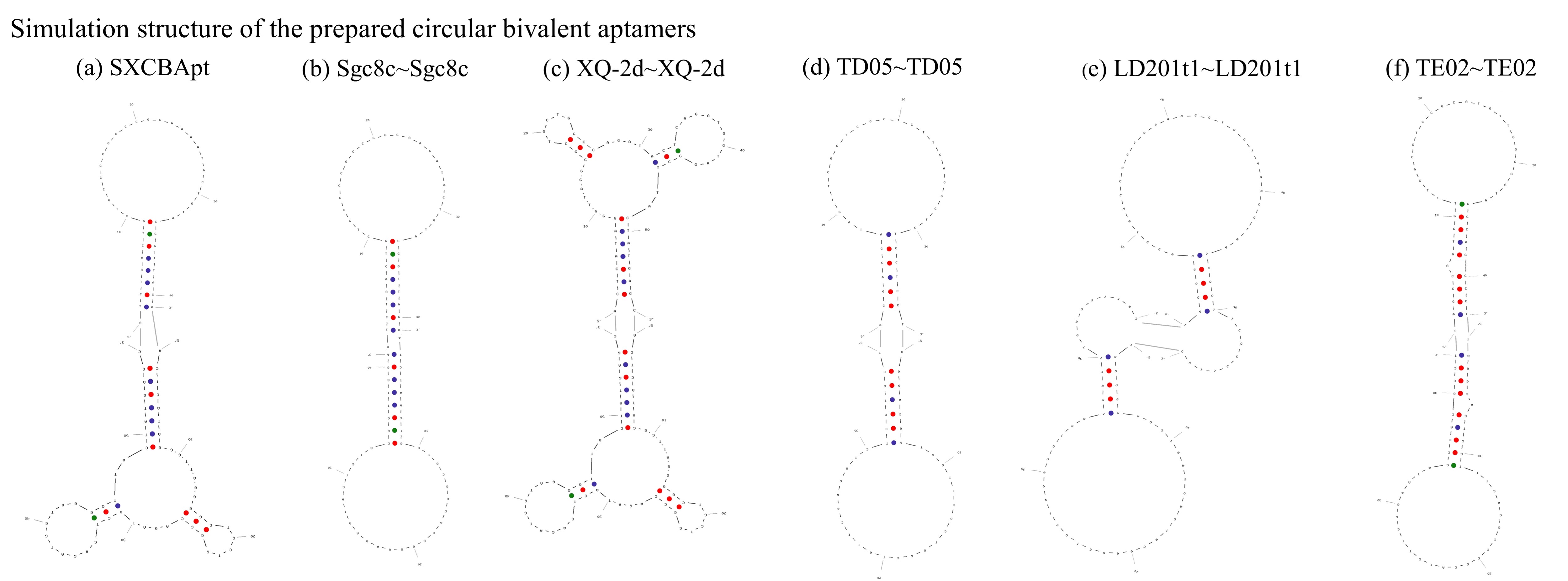


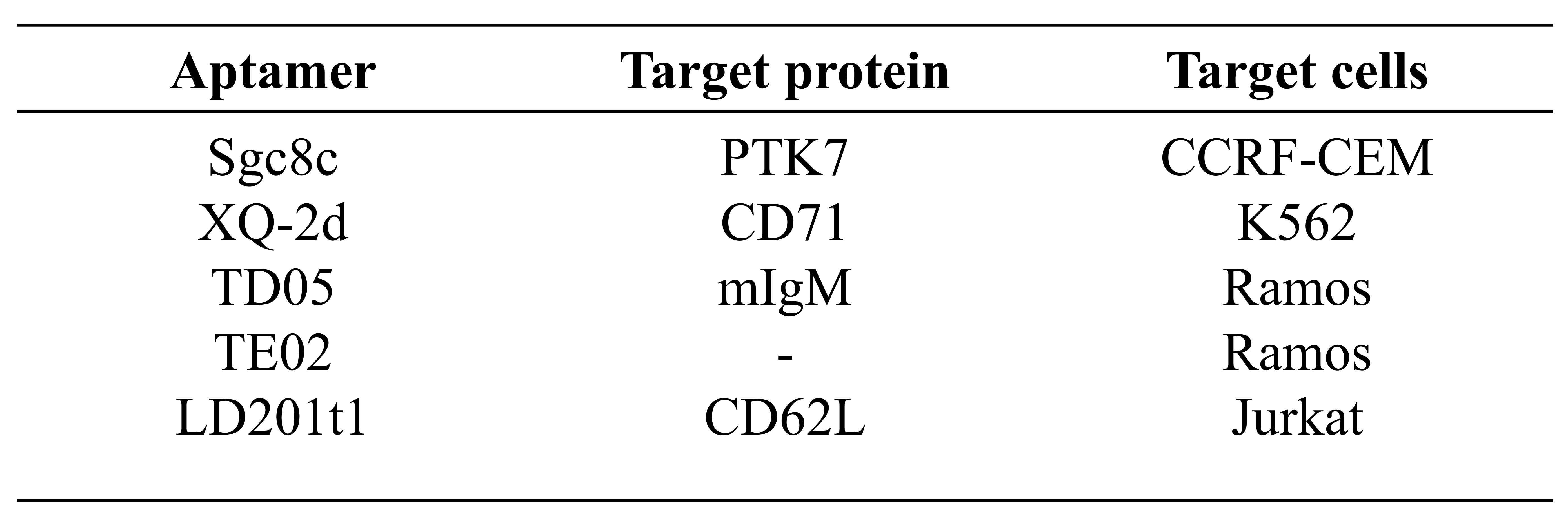


**Figure S4**. The simulation structure of, monovalent aptamers, the prepared circular bivalent aptamers, and their corresponding targets.





**Figure S5.** Calculated dissociation constants (Kd) of monovalent aptamers and the synthesized CBApts for their corresponding target cell lines.


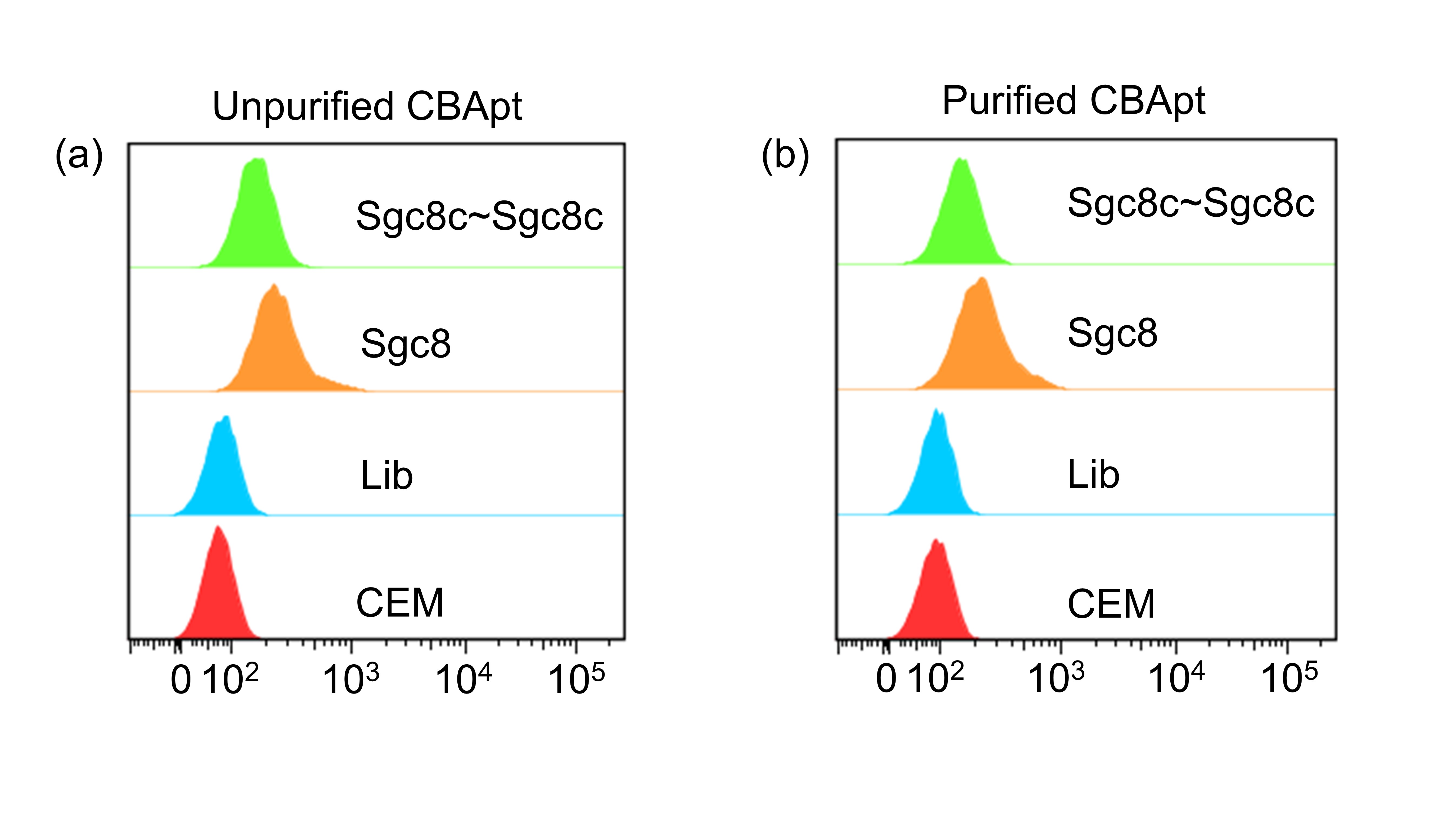


**Figure S6.** Effect of purity on the internalization ability of the CBApt. (a) the internalization ability of CBApt before purification. (b) the internalization ability of CBApt after purification.


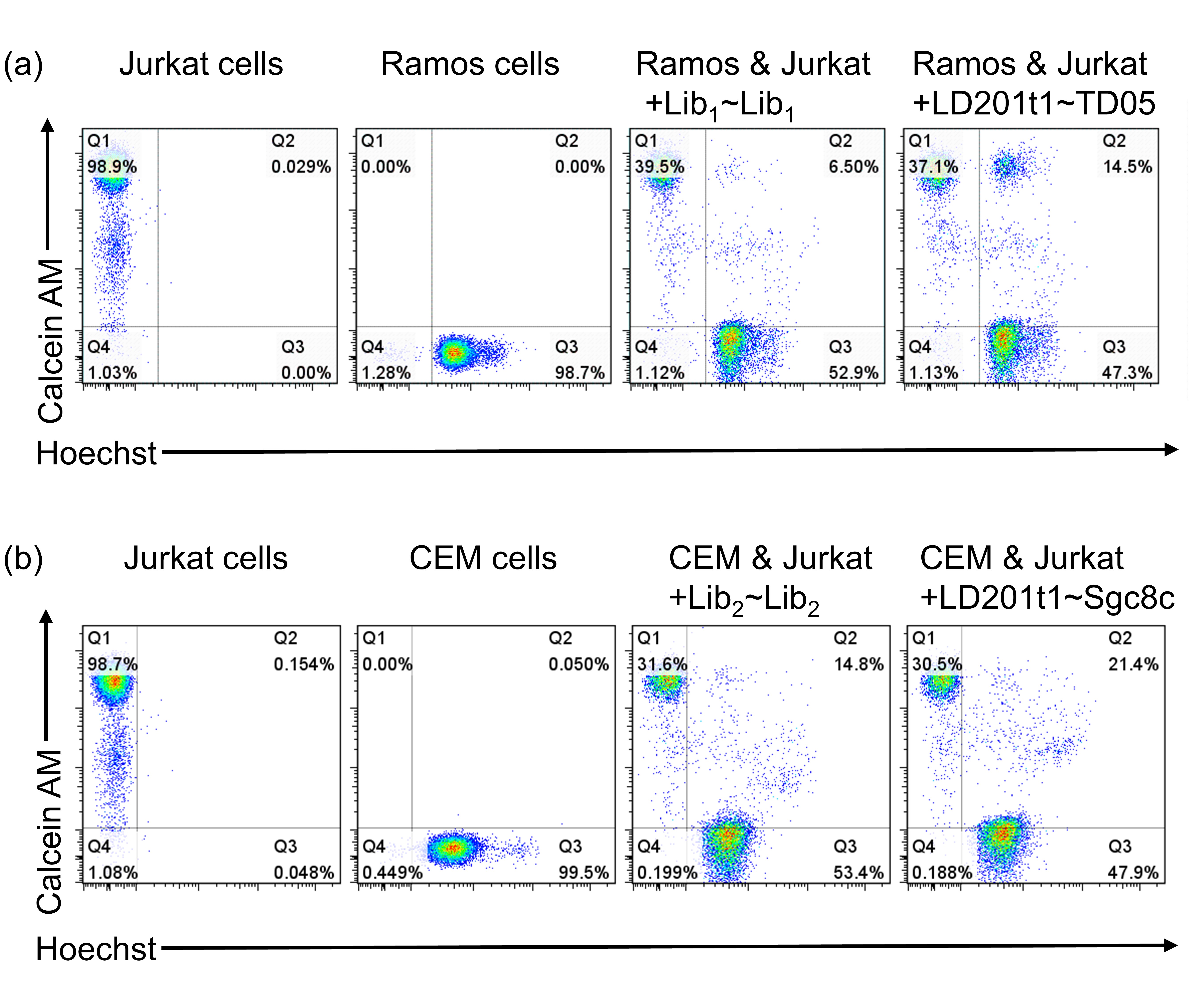


**Figure S7.** Flow cytometry analysis of junctional cell-cell complexes after a mixture of (a) Ramos cells and Jurkat cells were treated with LD201t1~TD05 or Lib_1_~Lib_1_ sequences; (b) CCRF-CEM cells and Jurkat cells were treated with LD201t1~Sgc8 or Lib_2_+Lib_2_. Jurkat cells were stained with Calcein AM, Ramos cells, and CCRF-CEM cells were stained with Hoechst.


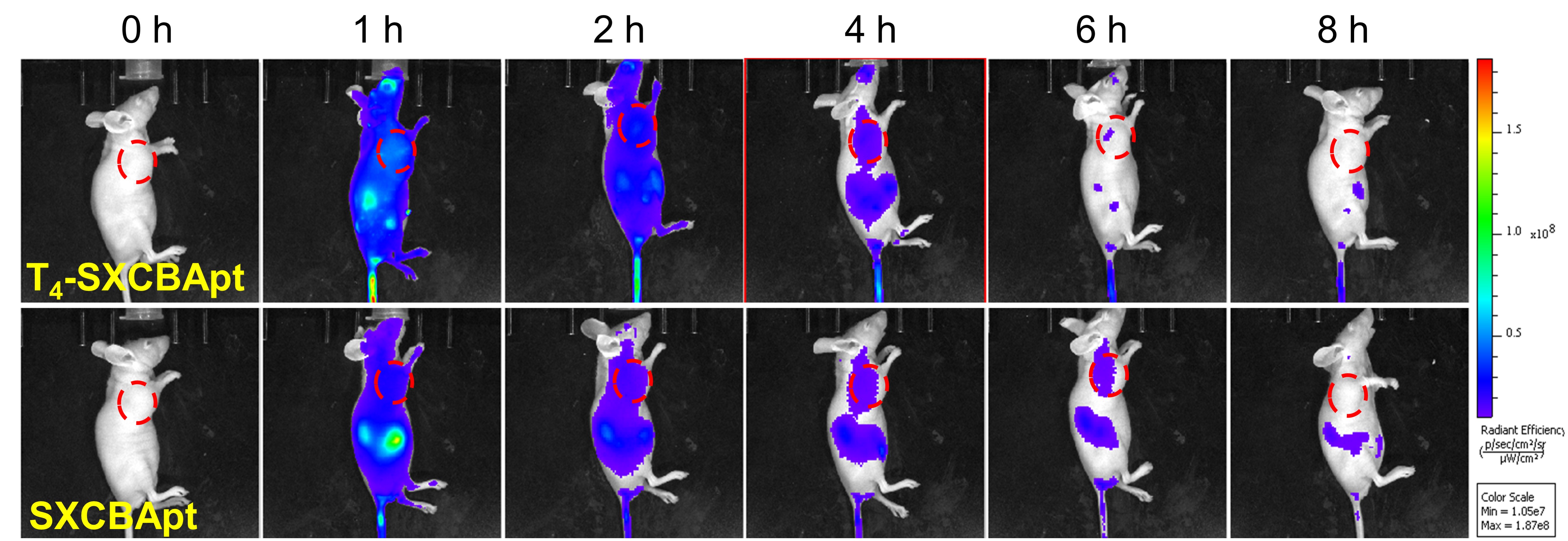


**Figure S8.** Comparing the fluorescence intensity of SXCBApt and T4-SXCBApt in tumor tissues using *in vivo* imaging technology.
